# Breaking Functional Connectivity into Components: A Novel Approach Using an Individual-Based Model, and First Outcomes

**DOI:** 10.1371/journal.pone.0022355

**Published:** 2011-08-01

**Authors:** Guy Pe'er, Klaus Henle, Claudia Dislich, Karin Frank

**Affiliations:** 1 Department of Ecological Modelling, UFZ - Helmholtz Centre for Environmental Research, Leipzig, Germany; 2 Department of Conservation Biology, UFZ - Helmholtz Centre for Environmental Research, Leipzig, Germany; 3 Biodiversity Conservation Lab, Department of Environmental Studies, University of the Aegean, Mytilini, Greece; University of Bristol, United Kingdom

## Abstract

Landscape connectivity is a key factor determining the viability of populations in fragmented landscapes. Predicting ‘functional connectivity’, namely whether a patch or a landscape functions as connected from the perspective of a focal species, poses various challenges. First, empirical data on the movement behaviour of species is often scarce. Second, animal-landscape interactions are bound to yield complex patterns. Lastly, functional connectivity involves various components that are rarely assessed separately. We introduce the spatially explicit, individual-based model *FunCon* as means to distinguish between components of functional connectivity and to assess how each of them affects the sensitivity of species and communities to landscape structures. We then present the results of exploratory simulations over six landscapes of different fragmentation levels and across a range of hypothetical bird species that differ in their response to habitat edges. i) Our results demonstrate that estimations of functional connectivity depend not only on the response of species to edges (avoidance versus penetration into the matrix), the movement mode investigated (home range movements versus dispersal), and the way in which the matrix is being crossed (random walk versus gap crossing), but also on the choice of connectivity measure (in this case, the model output examined). ii) We further show a strong effect of the mortality scenario applied, indicating that movement decisions that do not fully match the mortality risks are likely to reduce connectivity and enhance sensitivity to fragmentation. iii) Despite these complexities, some consistent patterns emerged. For instance, the ranking order of landscapes in terms of functional connectivity was mostly consistent across the entire range of hypothetical species, indicating that simple landscape indices can potentially serve as valuable surrogates for functional connectivity. Yet such simplifications must be carefully evaluated in terms of the components of functional connectivity they actually predict.

## Introduction

Landscape connectivity is one of the key factors determining the viability of populations and species in fragmented landscapes [1]–[3]. Consequently, an increasing number of empirical and theoretical studies attempt to predict patterns of landscape connectivity [4]–[7], and the literature proliferates with indices that could potentially describe and summarize it [8]–[10]. Nonetheless, there is still no consensus on how to measure landscape connectivity [11]. Predicting ‘functional connectivity’, i.e., whether a patch or landscape actually functions as connected from the perspective of a population or a species presents particular challenges. This is because functional connectivity is the outcome of complex interactions between individuals, populations, and landscapes [12]–[16] and it cannot be predicted without considering multiple factors: structural connectivity, the biology of species, the location and status of individuals, the decision-making processes that guide their decisions, and the factors that facilitate or impede their movement through landscapes. While empirical data on species' biology and habitat requirements is often obtainable, the behavioural responses and decision-making processes have only been studied for a handful of species and in specific contexts [17]–[22]. Hence, there is a need for tools that allow estimating functional connectivity for various species in a multitude of landscapes, despite the paucity of empirical knowledge on species' behaviour.

Another challenge in studying functional connectivity arises from the fact that ‘functional connectivity’ emerges from various movement components, each of which contributes to connectivity and thus to the functioning of populations, species, and communities in fragmented landscapes. Thus far, however, connectivity studies have either addressed *specific* components of connectivity, particularly dispersal, or they have analyzed connectivity as an *overall* outcome of the various movement processes (including foraging, mate searching, dispersal etc.) but rarely separating them. The latter approach is mostly taken by models designed to supply biologically realistic predictions, such as population viability analyses (PVAs; e.g. [23]–[25]). Recently, Mueller and Fagan [26] suggested that studies of animal movements must distinguish between different types of movement within their respective context. Following the same line, we suggest that an advanced understanding of the link between functional connectivity and species' sensitivity to landscape structures requires making at least three distinctions between components of functional connectivity: i) everyday movements differ from movements performed during dispersal; ii) during both everyday and dispersal movements, the movement pattern of individuals when moving between habitat patches can be either direct (gap-crossing) or more complex (e.g. random walk), depending on the capability of species to detect neighbouring habitat patches; and finally, iii) species may respond gradually or abruptly to habitat edges, some species avoiding the matrix while others penetrate into it. In the following we elaborate on each of these distinctions.

### Everyday versus dispersal movement

For many species, a conceptual distinction must be made between common everyday movements and rare dispersal behaviour [24], [25]. Everyday movements may involve multiple short-distance exchanges between habitat patches, to fulfil the individual's needs: food, shelter, mating, and reproduction etc. Dispersal, on the other hand, is a transient stage in an animal's life in which it may move through habitats that are unsuitable in terms of everyday needs. It involves different movement behaviour (higher correlation level, likely not returning to the same point) and involves greater distances [17], [27]–[31]. While functional connectivity during everyday movements may determine whether animals can maintain themselves in a fragmented landscape and therefore having immediate consequences on population dynamics, the effects of dispersal on (meta)population dynamics occurs over a longer and larger scale, via the rescue effect [32] or (re)colonization of habitat patches [33], [34]. Therefore, focusing on connectivity from the dispersal perspective only, may lead to overlooking important effects of functional connectivity on (meta)population dynamics.

### How to cross the matrix: random walk versus gap crossing

Having arrived at the edge of a patch, the probability that an animal will move to another patch can be simulated in various ways. One approach is to proceed with a per-step movement process – e.g., a random or correlated-random walk, as it was simulated up to the point of encountering the edge. In this case, if cell quality in the matrix is higher than zero, then animals can move into the matrix and between patches. A per-step simulation approach, where animals have to explicitly move through the landscape, is particularly useful when landscapes are heterogeneous and neighbouring patches are beyond the species' perceptual range: It allows accounting for the many decisions that animals make in response to landscape heterogeneity [35]. Another approach is to ‘relocate’ individuals in a single step, without explicitly accounting for the actual movement process through the matrix. In this case, it is often assumed that movements between patches are straight and their probability of occurrence can be calculated solely based on distance, permeability, and the associated costs (time, energy, mortality risk). This approach is comparable to situations where an animal can detect a neighbouring patch within its perceptual range and move directly to it. In forest ecosystems, such movements are often termed ‘gap crossing’ [36]–[38] – an important type of interpatch movement that strongly affects species' prevalence in fragmented landscapes [36], [38]–[42].

Connectivity models often simulate movements across the matrix either in a per-step approach or by relocating individuals in a discrete event. But most likely, animals perform *both* types of movement depending on distance, the detectability of neighbouring patches, and the structure and heterogeneity of the matrix. Both random walks and gap crossing can therefore be regarded as *components* of functional connectivity, but the contribution of each to connectivity and to (meta)population functioning in fragmented landscapes, has yet to be determined.

### Response to habitat edges: gradual versus abrupt, avoidance versus penetration

Changes in species abundance near habitat edges are often termed ‘edge effects’ [43], [44]. Edge effects result from alterations in environmental conditions, vegetation structure and composition [45], [46], resource availability [47]–[49], and altered biotic interactions, such as predation and parasitism [50], [51]. Recent studies have shown that edge effects reduce both the quality of habitat patches and the functional connectivity between them [20], [43], [49], [52], [53]. Furthermore, the response of species to habitat edges may occur on both sides of the edge: some species may avoid edges, others may penetrate into the matrix, and yet others may prefer the ecotone, i.e., the transition area [45], [54]–[56]. Gradients in habitat quality near edges, or alternatively gradual responses of species to edges, are particularly relevant in landscape mosaics where habitat boundaries are abundant, and they have a substantial effect on connectivity [57]. Hence, consideration of the species' response to habitat edges – gradual versus abrupt, avoidance versus matrix penetration – is imperative for understanding animal movement, species persistence, and community structures in fragmented landscapes [44], [58], [59].

### Individual-based models

Individual-based simulation models (IBMs) provide an excellent framework for studying connectivity. They can be used for examining how animal-landscape interactions, at the individual and local level, translate into higher-scale ecological and spatial patterns [60], [61]. A ‘pattern-oriented modelling’ approach, where model outputs are confronted with various observed patterns [62], [63], allows IBMs to address cases where empirical knowledge is insufficient for linking individual-level processes to landscape-level patterns. Consequently, IBMs have been widely applied for studying connectivity and dispersal and assessing how dispersal affects the functioning of (meta)populations in heterogeneous landscapes [17], [24], [35], [57], [64]–[69].

This paper introduces the individual-based model *FunCon*, which enables analysing functional connectivity by separating it into components. It distinguishes everyday (home-range) movements from dispersal, random walks from gap crossing, and it takes gradual responses of species to (supposedly abrupt) habitat edges into account. Thereby, it can be used to assess how different movement behaviours and movement modes affect the response of species and communities to habitat loss and fragmentation.

The *FunCon* model was developed in order to understand how connectivity affects the abundance and distribution of birds in the Atlantic rainforest of South America, potentially using empirical data [39], [49], [70]–[75]. With only 12.7% of the original forest remaining, over 80% of which is made up of small and isolated patches [76], the Atlantic Rainforest is one of Earth's biodiversity hotspots [77], [78] and an area where connectivity likely plays a major role in determining species' abundance and distribution. Yet the model was also designed to address gaps in ecological theory. We were particularly interested in exploring whether connectivity enhances species' viability in fragmented landscapes, and whether simple indices of structural connectivity can be used to predict the functioning of species across landscapes. Thus, the *FunCon* model can be tailored to address both specific applied issues and more theoretical questions.

This particular study attempts to establish solid foundations for a systematic, component-based understanding of functional connectivity. We first present the model concept and structure following the ODD protocol (Overview, Design concept, Details [79], [80]). Then, we apply the model to address three main questions. First, how does consideration of the behavioural mode (everyday versus dispersal) or the way of crossing the matrix (random walk versus gap crossing) affect our prediction of functional connectivity across species (from edge avoidance to matrix-penetration) and landscapes (from fragmented to more clustered)? Second, given that a detailed approach for assessing functional connectivity is bound to yield complexity, do consistent patterns emerge across species or landscapes that could potentially be used to simplify predictions of functional connectivity? And third, how is functional connectivity affected by different mortality scenarios, where mortality risks are either directly related to habitat quality or only differ (discretely) between habitat types?

By addressing these questions, we demonstrate the importance of separating functional connectivity into components for understanding the responses of species to landscape structures. We then discuss the limitations of current approaches to study connectivity, the potential power of our suggested approach, and the range of applicability of the *FunCon* model.

## Methods

### Model description

#### Model purpose

The *FunCon* model is designed to analyze how animal-landscape interactions determine functional connectivity for multiple species and landscapes, and how functional connectivity, in turn, affects the functioning of species in fragmented landscapes. Additional aims are to (i) identify critical thresholds of fragmentation and habitat loss for different species; (ii) assess how different connectivity indices can predict these thresholds; and (iii) contribute to deriving rules of thumb for conservation in fragmented landscapes. Programmed with Delphi (‘visual Pascal’) and running on a ‘Windows’ environment, *FunCon* can be used not only by modellers but also by ecologists without programming skills. It features a user-friendly interface for parameterization, calibration and analyses, and is freely available online (www.ufz.de/index.php?en=21420).

#### State variables and scales

*Basic entities:* Individual birds are the basic entities of the model. Each bird is defined by its state (alive or dead, successfully established in its home range or floater), its initial (‘home base’), current, and previous locations. A range of additional information is saved during simulations (see *Outputs*). The model does not explicitly include higher level entities such as populations or species communities, neither does it explicitly consider differences between sexes.

*Landscape units:* The model runs over grid-based maps with a rectangular 8-neighbour system. Cells belong to a certain habitat type, in this study either forest or matrix. Clusters of neighbouring forest cells form ‘forest patches’, each of which is characterized by its unique ID, area, and perimeter. Additional information for each patch is gathered during simulations (see *Outputs*).

*Sources of abiotic and biotic information:* Abiotic information originates from habitat type and the distance of each cell to the nearest edge, jointly determining cell quality (values ranging from 0 to 1). In order to address gradual changes in habitat quality (as perceived by the species) across distance, from the forest interior into the matrix, we applied the following sigmoid function:
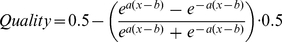
(1)where *x* is the distance along the ecotone (negative values inside the forest), *a* determines the slope of decay, and *b* determines the position along the ecotone (*x* axis) where habitat quality decays to 50% (the inflection point). The function, which is a derivation of the hyperbolic tangent [81], was chosen due to its flexibility, symmetrical structure, and the intuitive meaning of *a* (slope) and *b* (‘location’ of the curve). The formula determines the quality of each cell, either for home range or for dispersal movements, depending on the distance of each cell to the nearest edge. One may apply several decay functions to account for differential response of species to different matrix types, but in this study we focus only on one matrix type. The model does not consider other abiotic sources of landscape heterogeneity.

Among the various biotic interactions that affect home-range movements, we included three parameters: cell quality (which alters when a cell is occupied – see *Processes*), the maximum number of birds that can occupy a cell, and the maximum possible overlap between home ranges. The combination of these parameters allows density-dependence, both negative (territoriality) and positive (facilitation) to be included in the model. In the context of dispersal, intraspecific interactions are considered via the rules that instigate and terminate dispersal, as well as by offering several options for coupling the number of dispersers with the number of established birds. Interspecific interactions are not considered.

*Spatial and temporal scales:* For this study we used virtual landscapes produced by the landscape generator *G-RaFFe*, which Generates Roads and Fields for analysing Fragmentation Effects (see *Supplementary submodels*). Landscape extent was set to 335×335 cells and cell size was 30×30 m, in order to create landscapes equivalent to 10,000 ha – a typical area for landscape-level ecological analyses, particularly in the Atlantic rainforest [49], [72], [74], [75], [82], [83]. The landscape boundaries are reflective: if a landscape edge is reached, birds must reselect a direction. Thereby, we assume that the landscape edges behave similarly to the boundaries of a large hostile matrix, which tends to be avoided. To reduce potential artefacts caused by the landscape edge, the user can define the number of cells that function as a ‘buffer area’. In these cells, no bird releases occur.

In this study the home range size of birds was defined as 3 ha, based on the average home range of a small understory forest species in the Atlantic rainforest, as measured over a period of one month [73]. Simulation duration (number of steps) was determined in a way that ensured sufficient time steps for successful establishment (for further details see **Appendix S1**). The timescale for dispersal aimed to emulate a single dispersal event during an individual's life (e.g., juvenile dispersal). In this study, the maximum number of steps was 330 ( = a maximum distance of about 10 km). We note that these specific values represent only a limited number of species, but the qualitative results presented hereafter are largely independent of simulation duration (GP, unpublished data).

#### Processes and scheduling

**Figure 1:**
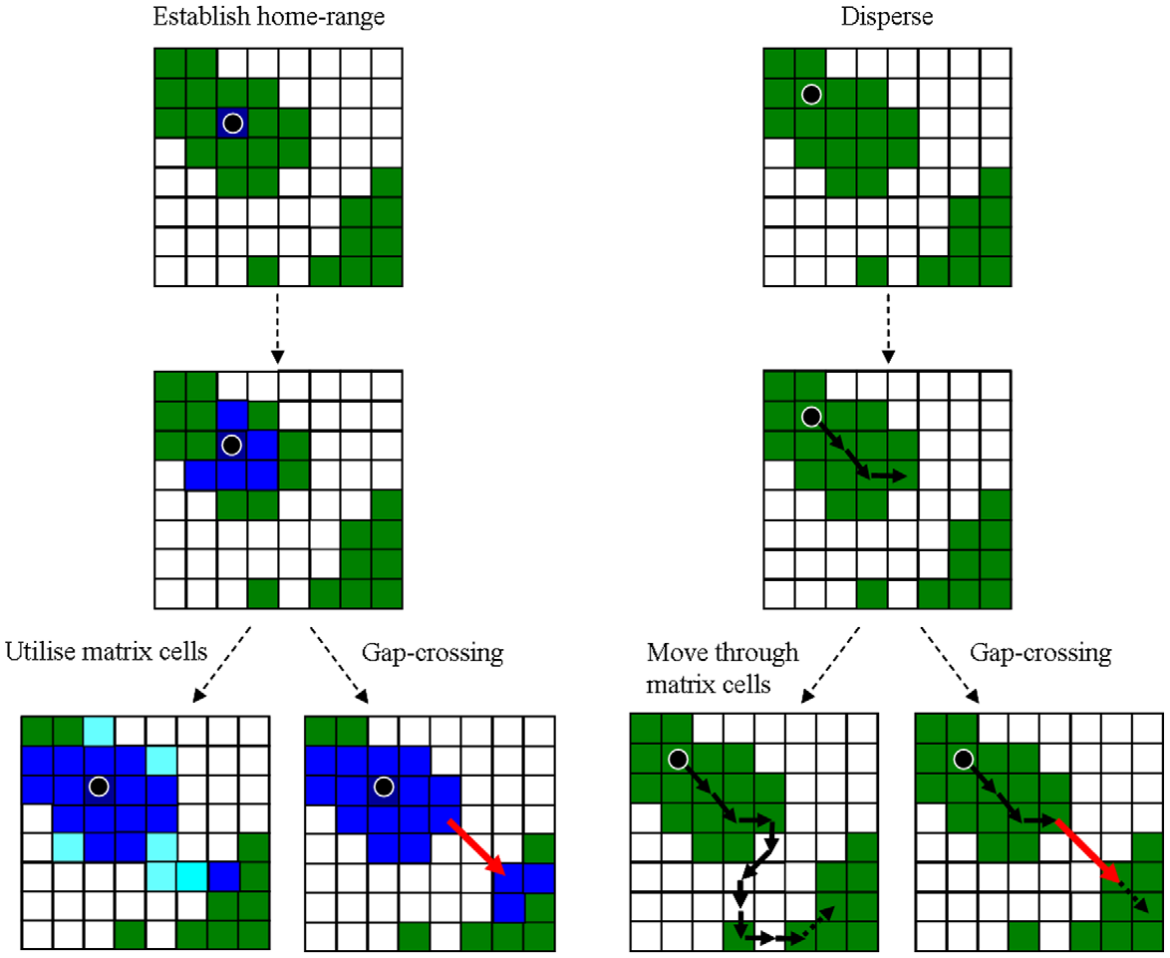
A conceptual scheme of the model's main processes. *Home-range* (left): A bird is introduced to a random forest cell (black spot) and occupies it as its “home-base” (dark blue); it moves randomly to any direction and occupies neighbouring cells (always returning to its home-base once occupying a new cell); in the expansion process it may occupy matrix cells (edge-penetration, left), thereby reaching other patches ( = connectivity) but with the cost of increasing the home-range size, or it may perform gap-crossing (right) and occupy cells in other patches without utilising matrix cells, (in which case the total number of cells until establishment may be smaller). Random-walk penetration into the matrix and gap-crossing are not mutually exclusive. *Dispersal* (righ): birds are introduced randomly into patches (not necessarily at the location where home-base are located), and move one step at a time – none of the cells is occupied. When reaching edges, birds may continue through the matrix (left path) or move between patches by means of gap-crossing. Simulation halts when arriving at a suitable patch (see text), or individuals may continue depending on a predetermined number of steps (dashed arrow).

*Home-range* (everyday) movements are simulated by distributing birds randomly in patches and then allowing them to establish home ranges (see **Figure 1** and flow chart in **Figure S1**). The birds ‘accumulate’ cells as part of their home range, each cell contributing to their effective home-range size according to cell size×quality. The movement of the birds within habitat patches is modelled as a correlated random walk with self-avoidance. Birds start at their home-base cell and move through their home range, one cell at a time, either moving forward in the same direction (probability = 0.5) or choosing from their seven immediate neighbours (eight minus the last one visited) according to their qualities. A bird can only move through cells that belong to its home range. Once it reaches a cell that is not yet part of its home range, it will take over the cell with a probability that equals cell quality or choose an alternative direction. If a cell is taken over, it contributes to the bird's effective home-range size, the quality of the cell can be recalculated and the bird is then relocated again at its home base to restart the search for the next cell to occupy. The option of altering cell quality allows competition or facilitation to take place by affecting the next birds that might attempt to take over the same cell, but this option was not taken in this study. The process of taking over cells reiterates until the bird's effective home range size surpasses its requirement (successful establishment), reaches the maximum number of steps (establishment fails), or dies (due to per-step, cell-dependent mortality). A bird has a limited area it can occupy (maximum number of cells). If it reaches this number without fulfilling its needs, establishment fails. The difference between the home-range requirement (which must be achieved through the effective area accumulated) and the maximum home-range size (measured only by area) determines the birds' flexibility to expand their realized home-range sizes in response to fragmentation, reduction in habitat quality, or competition.

We note that the overall process is not intended to mimic a real process of home-range establishment but to emulate the movement patterns that typify central place foraging, while forming relatively circular home ranges on the process.

Each of the birds is released and attempts to establish a home range sequentially. This sequence allows established birds to dominate over newly introduced ones. Birds that fail to establish a territory are removed at the end of the simulation (or registered as ‘floaters’), and their home-range movements are not considered as contributing to connectivity.

*Dispersal* is modelled separately from home range, using a separate set of parameters and a distinct habitat suitability map. The movement procedure is a correlated random walk, where animals start at random locations in forest patches and move through the landscape by choosing from their immediately neighbouring cells according to quality (see **Figure 1**). The dispersal process ends if a bird arrives at a suitable patch other than the patch of origin (successful establishment), dies (due to per-step, cell-dependent probability – dispersal fails) or reaches the maximum number of steps (dispersal fails). For other options of terminating dispersal or linking dispersal simulations with home range ones, see **Appendix S2**.

#### Sub-processes: response of birds to forest edges

During both home range movements and dispersal, animals can cross the matrix in two ways. The first occurs during the **random-walk** process: if habitat suitability allows it, animals may penetrate into matrix cells and move through them, one at a time, until potentially reaching another patch. We define this process as ‘random-walk penetration’ into the matrix. The abovementioned correlation factor (probability of continuing onward in the same direction), which is dominant over the response to cell qualities when moving within habitat cores, is cancelled in cells where the gradual edge effect is present (sigmoid function at values between 0.99 and 0.01), since it is unlikely that a correlated walk is dominant over the response to habitat edges or ecotones.

An alternative process, which may occur when birds are located at forest edge cells, is **gap crossing**. When a bird is situated at a forest edge, it knows the distance and the direction to the nearest non-self forest cell. The calculated probability of individuals to cross the gap decays with growing distance between patches, following the sigmoid function described above (hyperbolic tangent, *equation 1*), albeit with independent parameters. If the probability to cross exceeds a randomly drawn value, the cell on the other side is added as a ‘ninth neighbour’ (with the weight of its cell quality), additional to the eight immediate neighbours of the bird's locality, and it can be chosen by the bird according to the weights of the other eight cell qualities. Gap crossing and random-walk penetration are not mutually exclusive – an individual can perform both (unless edges are completely avoided, in which case gap crossing cannot take place). An implicit assumption of this approach is that the bird knows the quality of the target cells.

If a bird returns to an edge-location where it performed gap crossing earlier, its probability to perform gap crossing will not be calculated again. Instead, the bird could choose this cell as one of the ‘nine neighbours’ and move into it based on quality alone. Mortality risk, however, is always calculated according to distance. This rule follows empirical observations indicating that birds [40, M. Hansbauer, unpublished data] and other animals (e.g. geckos [84]) that establish territories over more than one patch perform gap crossing regularly, but likely not without costs [85].

An important distinction between gap crossing and random-walk penetration into the matrix is the different costs involved. While gap crossing involves only one movement step, random-walk movement through the matrix requires multiple steps (and therefore potentially higher mortality risk). In the case of home-range movements, an additional cost of random-walk penetration is the acquisition of low quality matrix cells in order to proceed through them into another habitat patch, whereas gap crossing allows ‘skipping’ the matrix cells and acquiring higher quality cells – requiring an overall smaller number of cells to fulfil the individual's requirements (cf. **Figure 1**). Consequently, random-walk penetration into the matrix bears a higher risk of reaching the home-range size limitation and therefore failing to establish territories.

#### Design concept

*Emergence:* Spatial patterns of connectivity, as well as the abundance and distribution of individuals across the landscape, are not imposed on the model but emerge from the specific animal-landscape interactions. Consequently, a pattern-oriented approach can be applied for model assessment, and model outputs can be related to observed patterns of abundance and distribution. Moreover, since establishment failure or success are explicitly considered, they provide additional ‘currencies’ for assessing how connectivity affects the functioning of species in fragmented landscapes.

*Sensing and interactions:* The movement of birds and their response to their surroundings depends primarily on information about the immediate 8-cell environment. Larger-scale effects on the birds' movement include edge effects on cell quality and knowledge of the distance and direction from each forest edge cell to the nearest neighbouring patch (see **Appendix S2** for details).

*Model outputs:* The model provides three main types of output: abundance of birds at the home-range stage, functional connectivity due to home-range movements, and functional connectivity due to dispersal. Outputs are provided for individuals, forest patches, and the entire landscape. For some of the visual outputs produced by the model see *Results*.

#### Model details

During *initialization* of home-range simulations, the model uploads a map, receives the species-specific input parameters, calculates a suitability map, and attempts to place birds in the landscape. The birds are introduced randomly, and in this study birds that are placed on non-forest cells are automatically removed. Birds that fall inside forest patches are introduced with a probability that equals cell quality, and only these can attempt to establish home ranges. As a result, the density of individuals at simulation initialisation reflects the effective habitat cover of any given landscape, edge effects included (see *Results*).

In this study the initial number of dispersers was set to equal the number of birds that successfully established a home range (hence assuming equality between population size and the number of dispersers). We also determined that dispersal only starts in patches where at least one bird has previously established a home range (albeit in a random location within these patches). This is done to prevent circumstances where dispersal can start in patches that are unlikely to serve as dispersal sources.

The model uses several groups of *input parameters*: The response of species to conspecifics; habitat requirements during home range and dispersal; parameters of decay in habitat quality at ecotones and decay in gap crossing probability with distance (for home ranges and dispersal); initialization of home range and dispersal; and general simulation inputs (simulation duration, perceptual range, landscape etc). A list of all parameters and values used within this study appear in **Table 1**. For detailed explanations of the parameters see **Appendix S2**.

**Table 1:** List of input parameters and their range.

Input parameter	Parameter type/range/units	Values in this study
**Response to conspecifics**		
Max. birds per cell	Integer (up to 20)	3
Max. overlap home ranges	Single (percent)	100
Change in quality of used cells	Proportion (change in quality)^a^	0
**Habitat requirements (home)**		
Quality forest	Single (0 to 1)^b^	1
Quality non-forest	Single (0 to 1)^b^	0
Min. home-range size	Single (ha)	3
Max. home-range size	Single (ha)	5
**Habitat requirements (dispersal)**		
Quality dispersal forest	Single (0 to 1)^b^	1
Quality dispersal non-forest	Single (0 to 1)^b^	0
**Behaviour at edges (home)**		
Activation of edge response	Boolean	Explored (yes/no)
X90 Edge effect (avoid/penetrate)^c^	Integer (m)	Explored (−200 to 150)
X50 Edge effect (avoid/penetrate)^c^	Integer (m)	Explored (−150 to 200)
Activation of gap crossing	Boolean	Explored (yes/no)
X90 distance gap crossing^c^	Integer (m)	Explored (0 to 150)
X50 distance gap crossing^c^	Integer (m)	Explored (50 to 200)
**Behaviour at edges (dispersal)**		
Activation of edge response	Boolean	Explored (yes/no)
X90 Edge effect (avoid/penetrate)^c^	Integer (m)	Explored (−200 to 150)
X50 Edge effect (avoid/penetrate)^c^	Integer (m)	Explored (−150 to 200)
Activation of gap crossing	Boolean	Explored (yes/no)
X90 distance gap crossing^c^	Integer (m)	Explored (0 to 150)
X50 distance gap crossing^c^	Integer (m)	Explored (50 to 200)
**Simulation initiation parameters**		
Num. birds to try introducing (home)	Integer	2000
Forest dweller	Boolean	Yes (home-base must be forest)
Num. dispersers equals…	Choice between 1) num birds placed; 2) num birds established; 3) fixed (Integer)	2
Num. dispersers	Integer (in the case of ‘fixed number’)	NA
Start only in ‘habitable’ patches	Boolean	Yes
Terminate only in ‘habitable’ patches	Boolean	Yes
Min. Num. of homes for ‘habitability’	Integer	1
Movement correlation factor	Single	0.5
**General simulation inputs**		
Number simulation repeats	Integer	50
Max. time steps home	Integer	3000
Max. time steps dispersal	Integer	330
Mortality scenario	Choice between1) per cell; 2) habitat type; 3) no mortality	Explored (1/2)
Perceptual range	Integer (m)	2000
Num. landscape cells from edge defined as ‘buffer’ (no releases can occur)	Integer	15
Landscape name	String	Six landscape maps

a **–** change in quality after a cell has been taken over by a bird;

b – basal quality when no edge effects considered.

c - determines the points along the ecotone (x axis) where the sigmoid function declines to 90% and 50% of its basal value – and, thereby, the slope of the decay function.

#### Supplementary submodels

The model utilizes seven raster maps and a separate list of forest patches that together provide all information that is necessary for the birds in terms of habitat type, distance to edges etc. These input maps are produced *a-priori* by two external submodels that extract the relevant information from any given land-use map (vector or raster). The first submodel was developed on an ArcMap template (ArcView 9.3) and the second, which calculates the distance and direction from each cell along the edge to the nearest non-self forest as means to enable gap crossing, was developed with Matlab. Both submodels are described in **Appendix S3**.

For the production of landscape maps for this study we utilised the landscape generator *G-RaFFe*, a process-based simulator which Generates Roads and Fields for analysing Fragmentation Effects. The model emulates the processes leading to habitat loss and fragmentation in arable lands: Namely, the construction of roads yields access to new regions, from which agricultural fields extend. The spatial patterns produced by the model are governed by the following parameters: the desired habitat cover, the number of roads, the maximum size of fields, and the maximal distance away from the roads, in which fields can occur (inverse of ‘road gravity’). For a given forest cover, increasing the number of roads, decreasing the maximum field size, or decreasing road gravity will enhance fragmentation. Apart from high flexibility and easiness of controlling the spatial patterns produced, ongoing tests of the model show that it successfully reproduces a wide range of spatial patterns in real landscapes (Pe'er et al., unpublished data). A ‘demo’ version of *G-RaFFe* is available online (www.ufz.de/index.php?en=21420).

**Figure 2:**
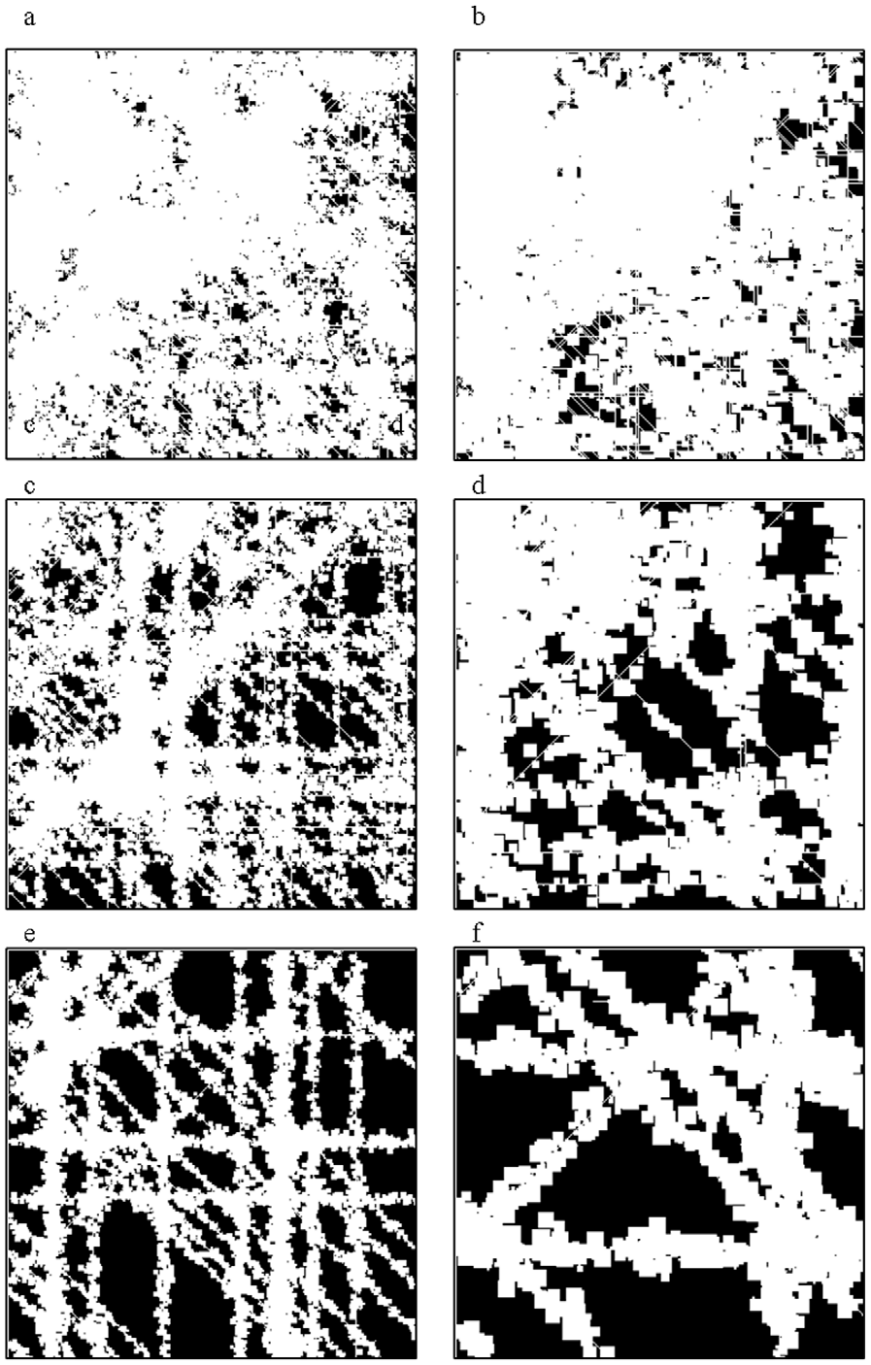
The six virtual landscapes used for simulations. We used landscapes with 10% (a+b), 30% (c+d), and 50% forest cover (e+f). Landscapes on the left were designed with a large number of roads and small fields (maximum field size 5×5 cells), yielding high level of fragmentation; landscapes on the right have a small number of roads and a maximum field size of 15×15 cells, yielding more clustered structures.

Simulations in this study were performed over six virtual landscapes produced by *G-RaFFe*, with 10, 30, and 50% forest cover (**Figure 2**). For each level of forest cover, we generated two landscapes: one with a high number of roads and small fields (highly fragmented), and one with a low number of roads and large fields (less fragmented, i.e. more clustered). Matrix heterogeneity was not considered in this study, therefore we used binary landscapes comprising only of forest and non-forest habitats.

### Simulations and analyses

In this study we performed three explorations, the specific parameters of which are summarized in **Table 2**. In the first exploration we altered the function that determines habitat quality during random-walk movements (i.e. producing edge effects), either during home range movements or during dispersal, from edge avoidance to matrix penetration. The slope of the sigmoid function was fixed while its location along the ecotone (x axis) was altered.

**Table 2:** Specific input parameters for the three main explorations performed in this study.

	Edge effects on connectivity – Random-walks	Compare random walks with gap crossing	Assess effects of connectivity on establishment success	
**Edge avoidance**	+		+	
**Random-walk penetration**	+	+	+	
**Gap crossing**		+		+
**Both random-walk penetration and gap crossing**		+		
**Parameter range of X50 (m)**	−150 to 200	50 to 200	−150 to 200	50 to 200
**Landscapes**	6	2: Least and most fragmented	6	6
**Mortality scenarios**	2	2	2	2
**Movement mode**	Home, Dispersal	Home, Dispersal	Home	Home

The slope of the decay function was determined by a fixed difference of 50 m between the points along the ecotone (x axis) where the sigmoid function of edge response or gap crossing declines to 90% (X90) and 50% (X50) of its basal value. The latter point is the inflection point of the function (‘b’ in *equation 1*).

In the second exploration we compared how random-walk penetration into the matrix, versus gap crossing, contributes to connectivity during home range and during dispersal. We thus altered the sigmoid function that determines habitat quality for random walks, the function determining the probability to perform gap-crossing, or both. Again, the shape of the function was fixed and only its position along the ecotone (the inflection point) was altered.

Lastly, in order to relate connectivity with abundance and distribution patterns across different landscapes, we assessed how the different edge responses (edge avoidance or penetration; gap crossing) affected the initial number of birds introduced into the landscape and their establishment success. Here, we only simulated home-range movements.

All three explorations were performed with two different mortality scenarios. In the first scenario, the per-step probability of mortality was inversely proportional to cell quality (hereafter, ‘cell-quality dependent mortality’), implicitly assuming that the quality of each cell represents the sum of costs, benefits and tradeoffs perceived by individuals (i.e., safe habitats are preferred over risky ones). In the second scenario we applied equal mortality risk across all cells within a given habitat type (henceforth, ‘fixed mortality per habitat type’), in order to emulate circumstances where the quality perceived by animals, and hence their movement decisions, do not fully match the ‘map of costs’. This could happen if animals either do not perceive the risks (e.g., [86], [87]), or if animals are forced to disregard them [88].

For simplicity's sake, we explored the same parameter range for both home range and dispersal simulations. Simulations were summarized using three connectivity measures for each of the two movement modes (home range or dispersal): the per-step, per-bird probability of interpatch movements (i.e. connectivity from the individual's perspective); the total number of interpatch movements (for all birds) as a measure of connectivity from the landscape and (meta)population perspective; and the proportion of patches in the landscape that received visits from other patches, as a spatial measure indicating how well the different patches are connected. In addition to these six connectivity outputs (three measures for each of the two movement modes), for the home range mode we also summarized the number of birds placed onto forest patches and the number of birds that successfully established home ranges.

## Results

### Example of results

**Figure 3:**
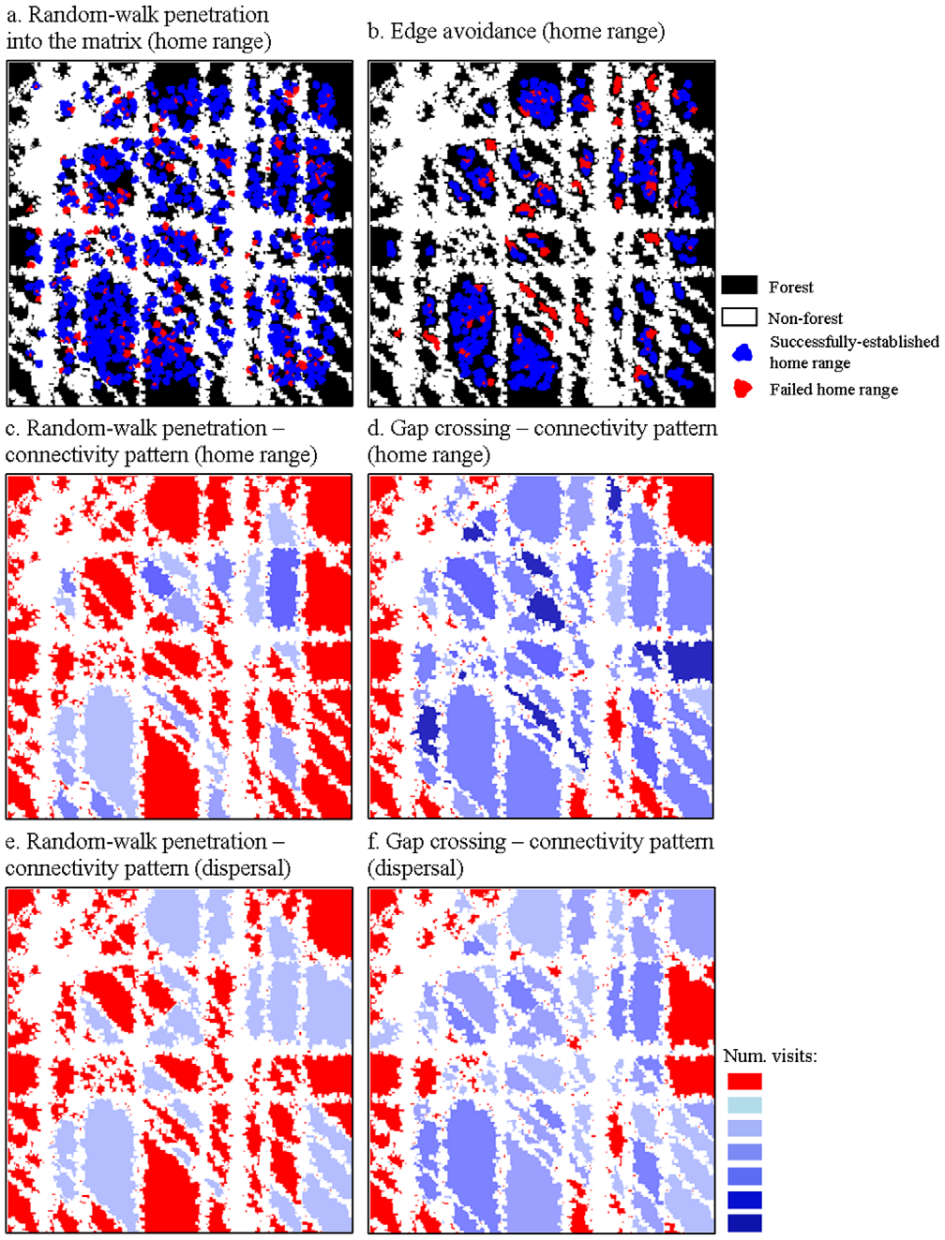
Examples of simulation outcomes. Forest areas in *a* and *b* are depicted in black, matrix in white, successfully established territories in blue, and red signifies cells where birds attempted to establish a territory but failed to do so; a) bird establishment success during home-range simulations, when birds penetrate into the edge by random walks (edge utilization; the 90% and 50% decay points of the sigmoid function were set to 100 and 150 m, respectively); b) bird establishment success under edge avoidance (90% and 50% decay points = −150 and −100 m, respectively); Using the same parameters as in (a), the remaining panels depict connectivity maps for c) random-walk penetration into the edge during home-range movements; d) gap crossing during home range movements; e) random-walk penetration into the matrix during dispersal movements and f) gap crossing during dispersal movements. Results are shown for a single simulation run over a virtual landscape with 50% forest cover (cf. Figure 2e).

**Figure 3** visualises outputs of the model from simulations over one exemplary landscape. In the first example, birds penetrate into the matrix by means of random walk. Introduced birds fail to establish home ranges in the smallest and most isolated patches (**Figure 3a**). Edge avoidance (**Figure 3b**) yields an even smaller initial number of birds, with an even larger proportion failing to establish home ranges - both in small and in medium-sized patches. Connectivity outcomes for both the home-range and the dispersal mode are exemplified through one simulation with random-walk penetration into the matrix (**Figures 3c,e**) and one with gap crossing movements (**Figures 3d,f**). For both movement modes, gap crossing increases the number of connected patches, and the overall number of interpatch movements is larger. Compared to home-range movements, dispersal movements distribute immigrants more evenly across the landscape (compare **Figure 3f** with **3d** and **Figure 3e** with **3c**).

### Analysis across a continuum of edge responses

**Figure 4:**
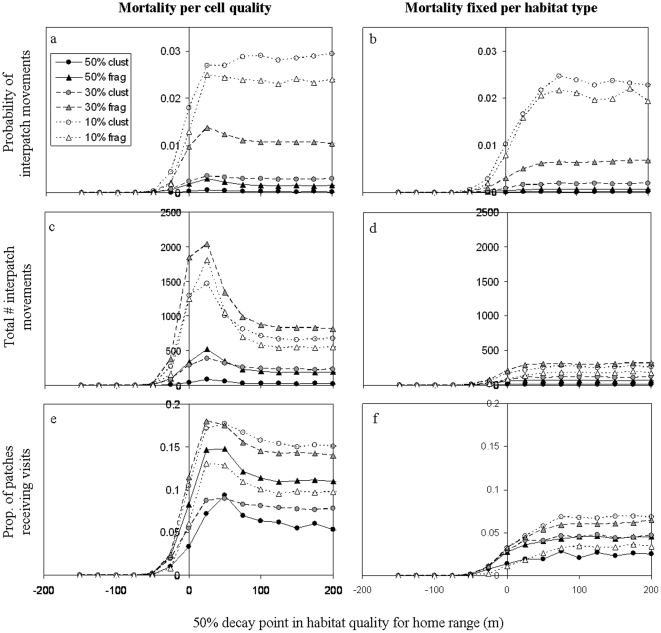
Systematic exploration of average connectivity during home-range movements, altering the edge response from edge avoidance to matrix penetration. *X* axis in this figure depict the 50% decay point of the sigmoid function (*b* in equation 1) determining habitat quality for random movements. Curves within figures represent the six landscapes. Figures on the left depict a scenario where mortality per cell is proportional to its quality, while figures on the right depict a scenario where mortality is fixed for each habitat type. The three lines of figures depict different measures of connectivity (simulation outputs): a,b) the probability for interpatch connectivity (interpatch movements per bird per step), c,d) the overall number of interpatch movements (for all birds and all movement steps), and e,f) the proportion of patches receiving interpatch moves from neighbouring patches. For all cases, the sigmoid function had the same slope (i.e., the 90% point is located 50 m deeper into the forest than the 50% point of decay). Values in the legend represent forest cover (%) and whether the landscape was more fragmented ( = frag) or more clustered ( = clust), see Figure 2.

**Figure 5:**
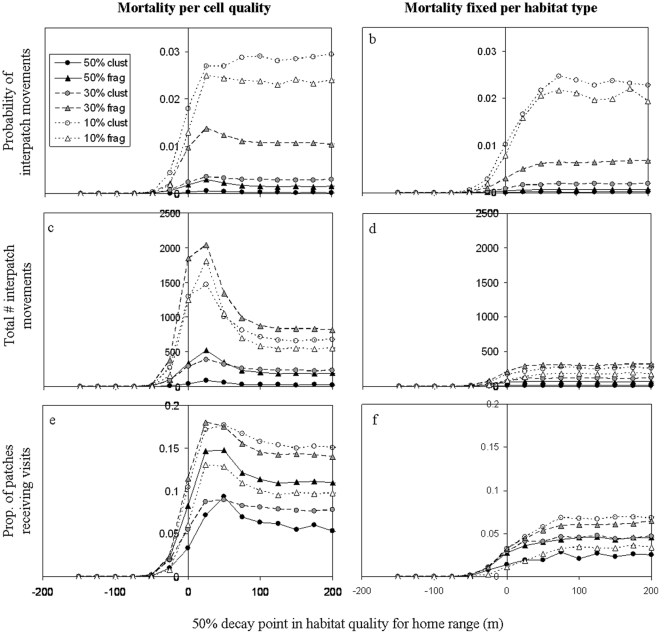
Systematic exploration of average connectivity during dispersal movements. For details see Figure 4.

Results in this section are based on a systematic alteration of the sigmoid function that determines habitat quality (and thus edge effects) for random-walk movements, from edge avoidance to random-walk penetration into the matrix, focusing first on home-range movements (**Figure 4**) and then on dispersal (**Figure 5**).

For all considered measures of connectivity and for both movement modes (**Figures 4** **,** **5**), altering the edge response of hypothetical species from avoidance to penetration has roughly resulted in one of two patterns: starting from zero when species avoid the edges, connectivity either increased monotonously with increasing edge-utilization until levelling off, or showed a hump-shaped pattern peaking at low penetration distance and then decreasing before levelling off.

Considering the probability of interpatch movements for **home-range simulations**, where mortality was proportional to cell quality, we found a monotonous pattern in the more clustered landscapes and a (very slight) hump-shaped in the more fragmented landscapes (**Figure 4a**). Comparing the curves for the different landscapes, the highest probability of interpatch movements was obtained in the second most fragmented landscape (10% habitat cover, small number of roads), followed by the most fragmented landscape and then decreasing as the level of fragmentation decreased. For a fixed mortality per habitat (**Figures 4b**), the probability of interpatch movements was reduced for all landscapes but the ranking order of landscapes remained the same as in the cell-quality dependent mortality scenario.

The overall number of interpatch movements in the landscape followed a clear hump-shaped pattern in all landscapes when mortality was proportional to cell quality (**Figure 4c**); fixed mortality per habitat resulted in a much more substantial reduction in the number of interpatch movements (**Figure 4d**), and the ranking order of the six landscapes in terms of connectivity has substantially altered (in the latter case, connectivity increased with the number of patches in the landscape). For the proportion of patches receiving visits from neighbouring patches (**Figures 4e,f**), the pattern was generally similar to the one obtained from the total number of interpatch movements (**cf.** **Figures 4c,d**), yet again the ranking order of landscapes has changed.

To summarize, in most cases, the shape of the curve was landscape-independent; where hump-shaped curves occurred in the cell-quality dependent mortality scenario, they were absent when mortality was fixed per habitat. In all cases, a fixed mortality per habitat type considerably reduced interpatch movements.

The ranking order of landscapes in terms of connectivity was only marginally affected by the edge response of species (only few crossing points of curves), and was largely maintained between mortality scenarios. However, we found a substantial change in the ranking order depending on the considered connectivity measure.

For **dispersal** movements (**Figure 5**) we obtained the same qualitative patterns, although for none of the connectivity measures did we obtain a strong peaking pattern as in **Figure 4**. As in the home range movements, all curves showed a levelling off pattern when mortality was fixed per habitat type. The ranking order of landscapes for dispersal movement was mostly preserved between the different mortality scenarios: Only 1–2 landscapes changed the ranking order. As another coherence with the outcomes of home-range movements, connectivity was consistently lower for fixed-per-habitat compared to cell-quality dependent mortality; this was most pronounced in the low total number of interpatch movements, due to mortality of most birds in the matrix (**Figures 5c,d**). Furthermore, as in the case of home-range movements, the ranking order of landscapes during dispersal has changed considerably between the considered connectivity measure (compare **Figures 5a,c,e with b,d,f**).

Yet some differences did emerge between the two movement modes. First of all, connectivity during dispersal movements was generally lower than connectivity due to home-range movements – in accordance with the smaller number of movement steps and the application of central-place foraging in the case of home-range movements. Second, the increase in connectivity with increasing edge penetration levelled off at slightly higher values of the sigmoid function compared to home range simulations, across all connectivity measures. Third, the ranking of landscapes between home-range and dispersal movement changed for all connectivity measures, most pronouncedly from **Figure 4e** to **5e** where for dispersal movements connectivity was highest in the two landscapes with 50% forest cover but home-range connectivity was maximal in landscapes with 10% and 30% forest cover.

Lastly, a pattern that was only observed for dispersal movements was a change in the ranking of landscapes along the edge response of species (crossing points of curves), both for the total number of interpatch movements and for the proportion of patches that received visits from neighbouring cells (**Figures 5c,e**), indicating an interaction between species' edge response and landscape.

### Random-walk penetration versus gap crossing

**Figure 6:**
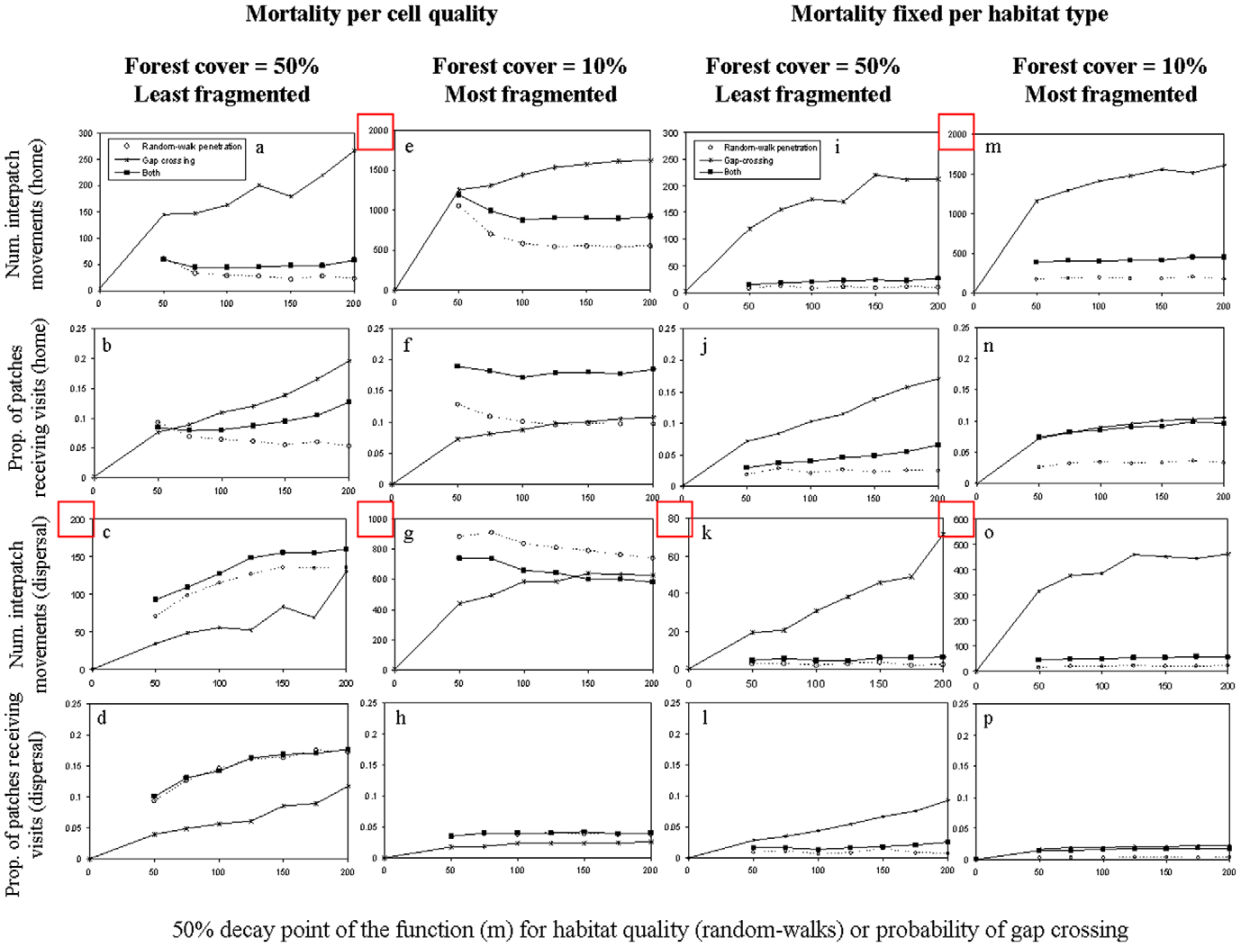
Comparing connectivity outcomes from random-walk penetration into the matrix, gap crossing, and a combination of both. We depict random-walk penetration into the matrix (open points, dashed lines), gap crossing (×, full lines), and the combination of both (full squares, bold lines), for the average total number of interpatch movements during home range (first line of subplots), the proportion of patches that received visits during home range (second line), the average total number of interpatch movements during dispersal (third line), and the proportion of patches that received visits during dispersal (lowest line of subplots). Results on the first and third column are for the least fragmented landscape (50% forest cover, clustered – cf. Figure 2f), results on the second and fourth column are for the most fragmented landscape (10% forest cover, fragmented – cf. Figure 2a). Results in the two left columns of subplots are for cell-quality dependent mortality, and in the two right-hand columns mortality is fixed per habitat type. X values represent the location of the 50% decay point of the sigmoid function (*b* in equation 1) along the interior-exterior forest ecotone. The point at X = 0 is depicted only for gap crossing but serves as reference both for no gap crossing and no usage of the edge. Note the different y-axis scales when the number of movements are counted (see red markings). Results for the probability of interpatch movements (per bird, per step) are not shown, as they were qualitatively similar to those obtained from the total number of interpatch movements.

We first describe the results of random-walk penetration versus gap crossing for a mortality that was proportional to cell quality (**Figure 6**, two left columns).

Connectivity increased monotonously with increasing the gap-crossing distance in the more clustered landscape, but tended to level off in the hyper-fragmented landscape, regardless of the connectivity measure used. Connectivity due to random-walk movements alone, as well as due to the combination of random-walk penetration with gap crossing, changed less steeply with distance. Whether random walk or gap crossing contributed more to connectivity depended on the landscape and the output measure examined: in some cases gap crossing contributed more (**Figures 6a,b,e**), in others it was random walks (**Figures 6c,d,g**). Note, however, that in the case of dispersal movements, random-walk movements always contributed to connectivity more than gap crossing (**Figures 6c,d,g,h**). Note also that only in two cases did the combination of random-walk penetration with gap crossing contributed more to connectivity than the singular edge-response (**Figures 6c,f**).

When mortality was fixed per habitat type (**Figure 6**, two right columns), connectivity still increased with increasing the distance to which birds were performing gap-crossing, but connectivity due to random-walk penetration was very low and almost independent of the penetration distance (constant along the x-axis) – for both movement modes, across all landscapes, and irrespective of the connectivity measure used. This low connectivity occurred due to mortality in the matrix when random walk through the matrix was applied. Accordingly, the combination of both gap crossing and random-walk movements yielded less connectivity than gap crossing alone.

### Relation between connectivity and abundance

**Figure 7:**
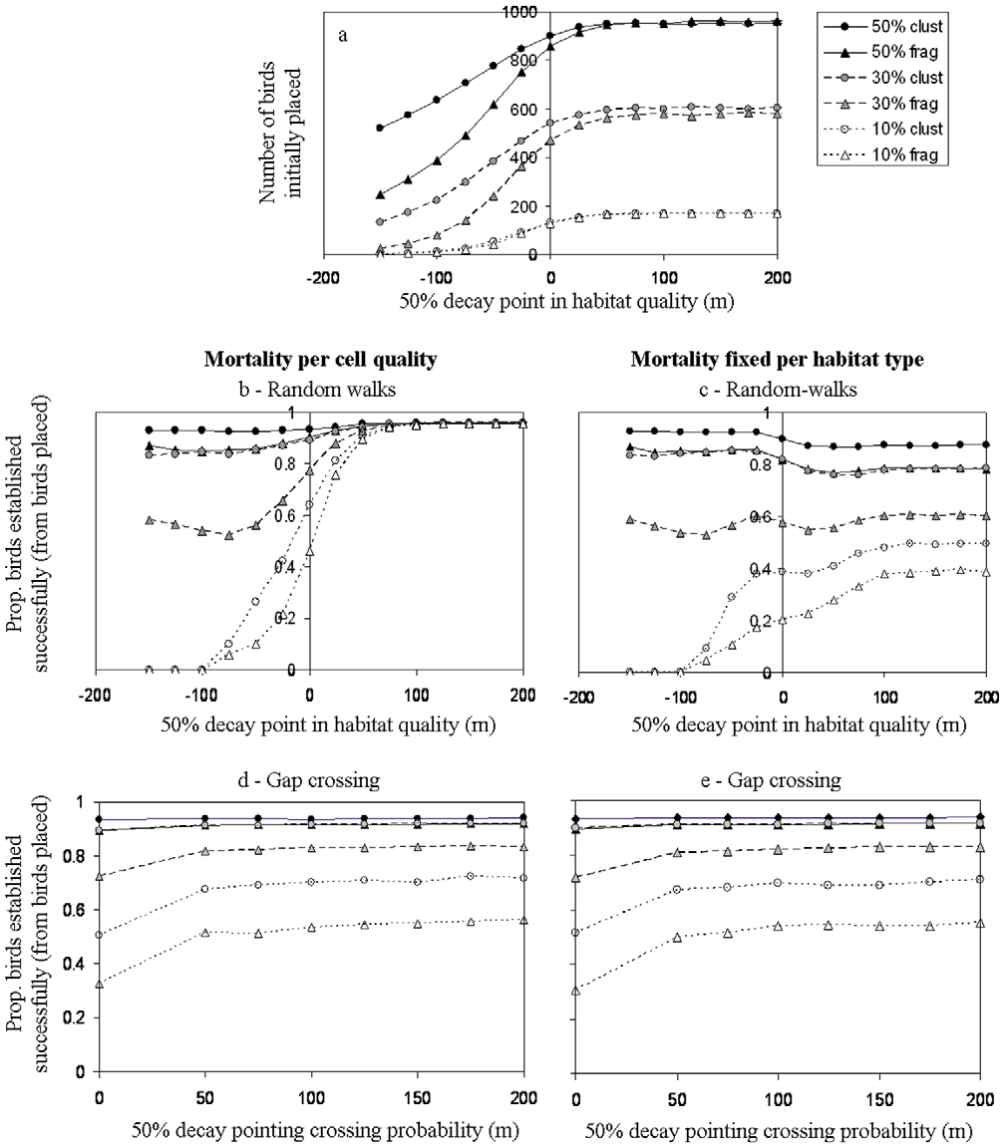
Assessment of the relation between edge response and the birds' success. a) effect of edge avoidance or penetration on the number of birds placed on each landscape when initializing home-range simulations; b–c) effect of edge avoidance versus random-walk penetration into the matrix on the success of establishing a home-range (out of the birds that were placed on each landscape), with b) a cell-quality dependent mortality and c) fixed mortality per habitat type; d–e) effect of gap crossing on establishment success (no edge response: habitat quality matches habitat type). Curves within each subplot depict the different landscapes, with legend values representing forest cover (%) and whether the landscape was fragmented ( = frag) or more clustered ( = clust), cf. Figure 2.

Altering the edge response from edge-avoidance to random-walk penetration into the matrix allowed a larger number of birds to be initially placed on the landscape (**Figure 7a**). As one may expect, the pattern of increase was landscape-specific because it is the size and shape of patches that determines how rapidly the effective forest cover falls with edge-avoidance. Notably, however, in the two landscapes with lowest forest cover (10%) we found only a marginal effect of the landscape structure, primarily because the maximal habitat availability was very low. Establishment success during simulations with cell-quality dependent mortality (**Figure 7b**) was close to 100% in the most clustered landscapes regardless of the response of species to edges. Yet in the more fragmented landscapes, edge usage became pivotal for establishment success, increasing it from zero to nearly 100%. When applying a fixed mortality per habitat, increasing edge usage had a positive effect on establishment success in the highly fragmented landscapes, but a negative effect in the less fragmented landscapes (**Figure 7c**). For all landscapes, penetration into the matrix beyond 100 m did not have any further influence on establishment success, due to the simulation properties (i.e., the fact that home-bases were always within forests and central-place (limited range) movements were applied).

The effect of gap crossing on establishment success (**Figures 7d,e**) was stepwise in nature: when gap crossing was applied it increased establishment success, but the effect was almost independent of distance that birds were willing to cross. This was true for all landscapes examined, the difference between landscapes being only in the extent of effect (i.e., smaller effect in the clustered landscapes where establishment success was anyway high). These results were independent of the mortality scenario applied.

## Discussion

Using the individual-based *FunCon* model, we assessed the contribution of different behavioural components to functional connectivity across a range of hypothetical species and landscapes. The explorations presented in this study demonstrate that functional connectivity depends not only on interactions between landscapes and species (e.g., alignment along the edge response) but also on the movement mode investigated (home range versus dispersal), the type of movement across the matrix (random walk versus gap crossing), the mortality scenario applied, and the choice of connectivity measure (in this case, the model output examined). By distinguishing between the components of connectivity we contribute to a better understanding of the relation between connectivity and the sensitivity of species to landscape structures. This is particularly essential when using indices of structural connectivity as surrogates for functional connectivity. Previous studies attempted to assess the predictive power of such indices [8], [11], [14], [89], [90] or offered advanced techniques to include important parameters, such as the biology of species, their niche profile, edge responses or response to habitat heterogeneity [16], [89], [91]. However, studies thus far have focused primarily on dispersal as a main process maintaining connectivity, and frequently utilised a single measure, often the probability of inter-patch movements, for quantifying connectivity. These simplifications have two main drawbacks. First, the everyday needs of individuals are often fulfilled through a narrower range of habitats, and the success of individuals in fulfilling their everyday needs have immediate effects on population dynamics. Consequently, it is likely that focusing on dispersal as a main process may not only lead to overestimating functional connectivity but also to underestimating its contribution to (meta)population dynamics in fragmented landscapes. Second, an important question has been overlooked thus far: namely, *what components* of functional connectivity are actually predicted by the various available indices. Our results clearly show that different measures of connectivity yield both qualitatively and quantitatively different results. In our case, the ranking order of landscapes in terms of connectivity differed between measures; a strong hump-shaped pattern of the curves (which was observed when mortality was inversely proportional to cell quality) was strongly evident when examining connectivity at the landscape level but far less noticeable and less consistent across landscapes when focusing on the probability of interpatch movements (compare **Figure 4c with 4a**); and finally, a reduction of connectivity when applying a fixed mortality per habitat type was far less substantial when examining the probability of interpatch movements (compare **Figures 4a,b** and **5a,b** with **Figures 4c–f** and **5c–f**). These results, as we elaborate below, reflect the fact that different connectivity measures bear important complementary information about functional connectivity. Therefore, the focus on a singular measure of connectivity may not only oversimplify connectivity but can even overlook patterns.

Our approach and the *FunCon* model allow overcoming these challenges. They enable testing the various landscape metrics against different components of functional connectivity, and assessing their true range of applicability. Thereby, we advance the emerging fields of behavioural landscape ecology [92] and movement ecology [93].

### Summary of main findings

#### Persistent patterns

Despite the complexity which may emerge from our detailed approach, our explorations revealed a number of persistent patterns that warrant special attention. One consistent pattern involved the ranking order of the landscapes in terms of connectivity (**Figures 4** **,** **5**). The fact that the curves rarely crossed each other means that the quality of a given landscape in terms of connectivity is generally well-maintained throughout the range of species, regardless of whether they avoid edges or penetrate into the matrix. This result suggests that simple measures of structural connectivity may in fact have considerable power in predicting functional connectivity, as long as one carefully examines what components of functional connectivity they actually predict. Note, however, that species×landscape interactions (i.e., crossing between curves) were most pronounced for the proportion of connected patches during dispersal (**Figure 5c**). This result strengthens the notion that the spatial patterns of dispersal may tend to be landscape-specific and therefore more complicated to predict (see also [57]).

Another consistent finding was the fact that gap crossing enhanced connectivity in a more monotonous manner compared to random-walk movements, and the outcomes of gap crossings were dependent primarily on distance but independent of the landscape or even the mortality scenario applied (**Figure 6**). On the other hand, comparisons between random walks and gap crossing in terms of their relative contribution to connectivity were highly dependent on movement mode (dispersal versus home range), landscape, alignment of the species along the axis of response to edges, and the connectivity measure used.

With respect to curve shapes along the different hypothetical species, we primarily identified two patterns: a hump-shape with peaking connectivity at short penetration distances, and a levelling-off pattern. In some cases the pattern was unaffected by the landscape in question (e.g. when examining home-range movements on the landscape level; **Figure 4**), and in all cases, a levelling off pattern occurred when mortality was fixed per habitat type (**Figures 4** **,** **5**). But the fact that two patterns emerged implies that the alignment of species along the interior-exterior gradient of edge response (avoidance versus penetration) cannot be intuitively interpreted in terms of connectivity: namely, ‘deeper into the matrix’ does not always translate into ‘more connectivity’. Here, further empirical data and models are still required across species and landscapes.

#### Less connectivity with deeper penetration into the matrix?

Somewhat counter-intuitively, most measures of connectivity indicated that matrix utilisation, particularly during home-range movements but to a lesser extent also during dispersal, yield a peaking level of connectivity when the hypothetical species penetrated short distances rather than deeply into the matrix (as long as mortality was proportional to cell quality). This result can be attributed to the fact that increased habitat availability within the matrix enabled birds to establish home ranges faster and therefore with fewer movements (see **Figure S2**). These results suggest that species that only penetrate to a limited extent into the matrix may need to move often between habitat patches. This does not mean that the landscape is necessarily ‘more connected’, but it does mean that functional connectivity is likely more critical for such species. These results may reflect biological realism. Animals in fragmented landscapes may need to increase their everyday movement distances in order to fulfil their requirements, resulting in higher costs but potentially also higher connectivity on the landscape level. Therefore, the consistency in patterns between the results obtained using the total number of interpatch movements and those obtained from the percentage of connected patches suggests that these measures bear important information on connectivity from the landscape perspective. By contrast, a more commonly used measure of connectivity such as the probability of interpatch movements ‘corrects’ for the varying numbers of individuals and movement steps, but it overlooks the question whether species *need* to or *tend* to move more in response to fragmentation. Hence, we suggest that the use of different connectivity measures – not only those presented in this study but also others, such as those originating from percolation-based approaches [94]–[97] or morphological spatial pattern analysis [98], may provide important information on complementary aspects of functional connectivity that are relevant at different scales.

Dispersal simulations showed a less marked hump-shaped pattern and only in some landscapes (**Figure 5a,c**). This can be explained by the fact that simulations halted when birds arrived at a habitable patch or died, meaning that the duration of dispersal depended on arrival at neighbouring patches rather than on patch quality. Our results for dispersal movements adhere more closely to simple measures of structural connectivity (the easier it is to cross the matrix, the higher the connectivity), but this only emphasizes the importance of considering both dispersal and everyday movements.

#### Mortality: not just a quantitative question

Instead of focusing on the question how high may be the costs of moving through different habitat types, our study offers a more qualitative perspective on mortality, asking what happens if animal decisions do not fully reflect the costs. Under the new ecological conditions imposed by habitat loss and fragmentation, animal decisions may not correspond optimally to food availability or risks. For example, animals may sometimes avoid edges despite the absence of risks, while others may penetrate into the matrix despite the associated risks –either because individuals are forced to ignore the risks, or because they are not aware of them – potentially resulting in ecological sinks [86], [87], [99]–[101]. Thus, we suggest that the two mortality scenarios applied in this study may be equally realistic, merely describing different circumstances or species.

Several important outcomes emerge when exploring these two mortality scenarios. First, the striking reduction in connectivity when applying a fixed mortality per habitat type, and the associated effect on establishment success, indicate that situations where mortality is disproportional to the movement decisions are most likely to inflict high sensitivity to fragmentation. Second, we found that a fixed mortality per habitat type always results with the same pattern along the avoidance-versus-penetration axis (i.e., levelling off). This means that the alignment of species along the interior-exterior gradient of edge response (avoidance versus penetration) may be indicative of connectivity at least in some circumstances, e.g. in our case when animal movement decisions do not fully match the distribution of costs. Lastly, when mortality risk was fixed per habitat, we found that random-walk penetration into the matrix yielded only marginal connectivity due to the mortality of individuals in the matrix, but connectivity due to gap crossing remained generally high (**Figure 6**, two right columns) – suggesting that fragmentation may favour species that avoid gaps completely (but then bear the consequences of complete isolation) or perform gap crossing, while selecting against species that move slowly through the matrix or penetrate into it despite associated risks. Note, however, that gap crossing cannot occur alongside edge avoidance, and therefore one might expect species that engage in gap crossing to be less sensitive to habitat fragmentation from the first place. Therefore, empirical data and models that explicitly consider how different costs affect the movement decisions taken by animals and the consequent balance between mortality and reproduction [85], [88], [102] may improve our understanding of the contribution of connectivity to population dynamics in fragmented landscapes.

#### The effects of edge avoidance or matrix penetration on establishment success

Another important pattern emerges when focusing on the establishment success of birds as means to understand how connectivity affects the functioning of species in fragmented landscapes. Exploring the range of edge responses from avoidance to penetration, we found that moving through the matrix may have a positive or negative effect on establishment success (relative to edge avoidance) depending on the type of mortality the species experiences, but it has a strong positive effect, regardless of the mortality scenario, in highly fragmented landscapes (**Figure 7**). These results strengthen the notion that connectivity has an imperative role for species' persistence in highly fragmented landscapes. Yet somewhat contrary to our expectations, gap crossing consistently had a smaller effect on establishment success than random-walk penetration into the matrix. These results can be explained by the fact that random-walk penetration was achieved by increasing matrix quality (and therefore habitat availability), while gap crossing involved a response to a fixed habitat availability. Further analyses are therefore required before generalizations can be made about the relative contribution of gap crossing and edge penetration to connectivity and to species' persistence in fragmented landscapes.

### Model application, limitations, and future prospects

The *FunCon* model can be used to study connectivity on various ecological levels and spatial scales, thus contributing to an emerging field of upscaling and downscaling in ecology [103]–[109]. This paper presented the outcomes of relatively qualitative explorations, over a limited number of landscapes. A more systematic analysis, taking a more quantitative approach and running on a wide range of landscapes, is currently undergoing, focusing on assessing the predictive power of various landscape metrics with respect to the different components of functional connectivity. We are further exploring the capacities of the model to predict empirical patterns at different ecological levels and spatial scales, from detailed analyses on the landscape level using radio-telemetry for three focal species [49], [71], [73], to larger-scale analyses of the response of bird communities to landscape structures using abundance data originating from point-counts [42], [83], [110]. These analyses have required analysing empirical data to separate habitat suitability in ‘habitat cores’ from the area affected by edges. We should stress the importance of such analyses for advancing a necessary integration between the topics of connectivity, edge effects, and habitat suitability ([59], Zurita et al. unpublished data).

Naturally, not all of the many factors that determine connectivity patterns were included in the model. In the following we provide two examples of elements that may be included in future model versions. First, one may wish to consider alternative movement strategies of animals in a landscape. Search strategies may include spiral movements or ‘loops’ [7], [111], flexible or shifting home ranges over time [73], [112]–[115], or learning processes that affect movement patterns (e.g. [116]). Second, the model does not consider alternative responses of animals to habitat boundaries [117]–[119]. Boundaries may be diffusive (as it currently is in our model), meaning that the decision to move through them depends only on quality or permeability of the matrix [120]; reflective, meaning that animals move back into the habitat core when an edge is met [121], [122]; or act as movement conduits, ‘channelling’ animals to move along the boundary [123], [124]. Such options were not included in the model due to their relative complexity, especially since empirical data are rarely available in order to parameterise them, but they are currently developed and could naturally gain from any advances in the knowledge of species' movement behaviour.

Finally, the model does not consider population dynamics and therefore the dynamic response of species to the landscape. A dynamic, multi-generation approach is critical for understanding how species and communities respond to landscape alterations and environmental changes across spatial scales [103]. Thus, current development of the modelling framework aims to incorporate these elements, in order to simulate the contraction or expansion of species in response to changes in land use and climate.

## Supporting Information

Appendix S1 **Determining simulation duration.**
(DOC)

Appendix S2 **Model inputs.**
(DOC)

Appendix S3 **Supplementary submodels for map preparation.**
(DOC)

Figure S1 **Flow chart of the process of home-range expansion.**
(TIF)

Figure S2 **Effect of edge response and landscape structure on the time to home-range establishment.** a) Number of time steps until home-range establishment, and (b) the number of interpatch movements divided by simulation duration (i.e., per step connectivity). Simulations are given for mortality per cell quality, for the six landscapes produced by the landscape generator *G-RaFFe*. Values in the legend represent forest cover (%) and whether the landscape was more fragmented ( = frag) or more clustered ( = clust).
(TIF)
