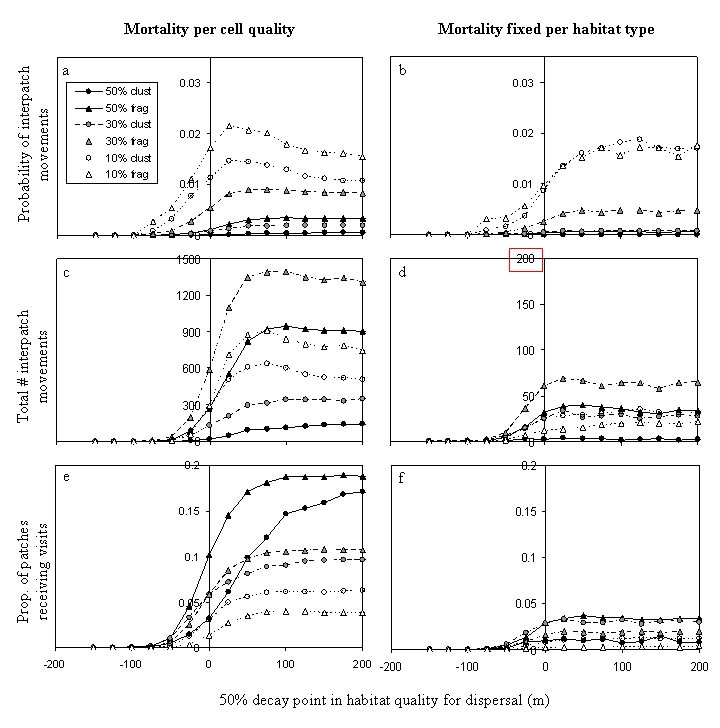# Correction: Breaking Functional Connectivity into Components: A Novel Approach Using an Individual-Based Model, and First Outcomes

**DOI:** 10.1371/annotation/68a211a3-1d14-4948-8486-53d4966429f6

**Published:** 2011-08-24

**Authors:** Guy Pe'er, Klaus Henle, Claudia Dislich, Karin Frank

The published Figure 5 is erroneously identical to Figure 4. The correct Figure 5 can be seen here: 

**Figure:**