# Supplementary material for: Breaking Functional Connectivity into Components: A Novel Approach Using an Individual-Based Model, and First Outcomes
Source: PLoS One. 2011 Aug 1;6(8):e22355. doi: 10.1371/journal.pone.0022355 (PMC3148224; doi:10.1371/journal.pone.0022355)
Supplement: Appendix S1 — Determining simulation duration. (DOC) [file pone.0022355.s001.doc]

**Appendix S1: Determining simulation duration**

The number of home-range steps in the model was in fact not driven from biological factors but adjusted in a way that ensures sufficient time steps for successful establishment of home ranges. When simulations are performed with different home-range sizes, one must account for the per-step mortality by scaling the number of time steps to the duration required for home-range establishment. This was not required in this study, but can be done by following the formula:

*TimeSteps* = *CellLength* × *0.63* × *Area ^1.835^*

These specific fitting parameters were found by trial and error. Their values can be explained by the fact that the movement diverges from a simple diffusion function ( = aX^2^), first because of their iterative return to the home centre, and second, because of the application of a correlation factor.

To determine the maximum duration of dispersal, we relied on the work of Bowman [1] who has shown that the mean dispersal distance is proportional to the breeding home-range size. The user can therefore determine a ratio factor *ρ* which relates the maximal number of dispersal time steps (*D*) to the home-range size of the species (*A*, in square meters), corrected by the map resolution (cell length *C*, in meters):

$$D=\rho\cdot(\sqrt{A/\pi})/C$$

In the absence of empirical data on dispersal time, one can refer to Bowman [1] or alternatively explore a range of values of *ρ*.

**Reference:**

1. Bowman J (2003) Is dispersal distance of birds proportional to territory size? Canadian Journal of Zoology-Revue Canadienne De Zoologie 81: 195-202.
