# Supplementary material for: Breaking Functional Connectivity into Components: A Novel Approach Using an Individual-Based Model, and First Outcomes
Source: PLoS One. 2011 Aug 1;6(8):e22355. doi: 10.1371/journal.pone.0022355 (PMC3148224; doi:10.1371/journal.pone.0022355)
Supplement: Appendix S2 — Model inputs. (DOC) [file pone.0022355.s002.doc]

**Appendix S2: Model Inputs**

The following paragraph summarizes all model inputs and explains their logic and role.

1. The *Response of species to conspecifics* during home-range movements is affected by three parameters that range from negative response (“territorial”), through no response (“flexible”) to positive response (“intra-specific facilitation”): the maximum number of birds in a cell, the maximum possible overlap between territories, and the change in cell quality if it is occupied by a bird.

2. The *niche profile* of the species defines the quality of cells for home-range, or for dispersal, for up to eight different habitat types. An additional parameter determines whether the species is forest-dwelling. In this case, birds that initially fall outside the forest are excluded.

3. *Behaviour at edges and gap crossing* are both modular (can be switched on or off). Both are determined by the same sigmoid function (equation 2), requiring two parameters per (non-forest) habitat: namely, the two distances in which the sigmoid function equals 90% and 50%, respectively, defining the decay in cell quality for edge utilization or the probability of crossing in the case of gap crossing. An additional Boolean parameter determines whether a change in forest ID, which indicates a gap smaller than cell size, is perceived by the species as a barrier. Lastly, the perceptual range of the birds defines the upper limit in which a neighbouring patch can be perceived and therefore where gap crossing can occur.

4. *Home-range parameters* include the minimal and maximal home-range size (both in units of area), and the maximal number of time steps for the simulation to run. As a default (based on preliminary simulations), the maximum number of home-range movements is set to *20·A·H^2^* where *A* is cell length and *H* is the minimum area requirement in ha. This is because the average time for establishing a home range follows the power law of *A·H^2^*, and hence the factor of 20 ensures that the number of movement steps does not limit establishment success, regardless of the home-range size used.

5. *Dispersal parameters* include, first, a choice between scenarios in terms of “who disperses”, namely a) floaters, b) constant proportion of the population (which would include all floaters if surpassing the percentage of floaters in the population); or c) birds that are randomly scattered in the landscape. The number of birds can equal a) the number of birds that were placed on the landscape during home-range initiation, b) the number of birds that established home ranges, or c) any other fixed number. Another Boolean input determines whether dispersers can start in any patch or only in habitable patches (where at least one home range was established). Next input determines the condition to stop dispersing: a) stop when reaching the maximum number of dispersal steps (or until death), b) disperse until reaching a new habitable patch, or c) disperse until reaching a habitable locality, and halt only if successfully establishing a home range there. Other parameters determine the maximum number of steps (which can be proportional to the home-range size, see equation 1), and the probability of performing a movement step in the same direction (to achieve a correlated random walk).

6. *Mortality*, either during home-range or during dispersal, is calculated automatically in order to adjust it to simulation duration. The inputs thus include only a selection between three scenarios: Mortality can be inverse-proportional to the quality of each cell in the landscape (meaning that avoidance is directly related to the actual risk), inverse-proportional to the general quality of each habitat type regardless of the distance to edges (meaning that penetration into the matrix bears a risk, while edge avoidance bears a missed-opportunity cost), or mortality can be set to zero. In the first two cases, the background per-step mortality (i.e., in forests) equals 1/(number of maximal simulation steps). In the case that habitat quality or cell quality equal zero, mortality is set to ×50 basal mortality.

7. *General simulation inputs* include the initial number of birds during home-range simulations, the number of landscape cells that are defined as edge and where birds cannot be placed during home-range or dispersal, and whether habitat quality is affected by an overall effect of patch size (regardless of explicit edge effects emerging from the sigmoid function). If activated, the area effect is determined again by the above-mentioned sigmoid function, the inputs of which are the sizes in which patch quality equals 90% and 50%, and the value of the "end value", namely the lowest value to which habitat quality can fall. Finally, for multiple simulations, one can receive all model parameters from an external input file, defining all input parameters, the number of simulations, which processes should run (i.e., home range, dispersal, or both), and what outputs should be stored.
