# Supplementary material for: Breaking Functional Connectivity into Components: A Novel Approach Using an Individual-Based Model, and First Outcomes
Source: PLoS One. 2011 Aug 1;6(8):e22355. doi: 10.1371/journal.pone.0022355 (PMC3148224; doi:10.1371/journal.pone.0022355)
Supplement: Appendix S3 — Supplementary submodels for map preparation. (DOC) [file pone.0022355.s003.doc]

**Appendix S3: Supplementary submodels for map preparation**

For simulations to run, we used two submodels that analyze any land-use map (on a raster or vector base) – one running on GIS (ArcView 9.3), the other one on Matlab [1] – that together produce nine files that are essential for the model. Seven of the outputs are raster matrices (layers of information) providing animals with the information they require for their decisions. These seven maps depict (1) habitat types, (2) forest patch IDs, (3) the distance of each cell to the nearest edge, (4) the direction from each forest cell toward the nearest edge, (5) the nearest non-forest habitat type (for each forest cell), and finally (6) the distance and (7) direction from any cell on forest edges to the nearest non-self forest edge. The additional two files enlist the forest patches (ID size and perimeter) and the raster extent.

The ArcMap model produces the following maps:

1. a land cover map (raster)

2. a map of the ID of each forest patch

3. the distance from each cell to the nearest edge, calculated once from cells inside the forest toward the outside, and then from the matrix into the forest edge. Distances inside forests obtain a negative sign, while positive values represent matrix cells.

For each cell *within* the forests, the GIS submodel also produces:

4. a map depicting land cover at the nearest non-forest cell neighbouring the forest, and

5. the direction toward that nearest matrix cell. Both maps derive from the “allocation” tool in ArcGIS. The direction to the edge is not used by the simulation model itself, but is used by the Matlab procedure described. Additionally to these five matrices, the GIS model also provides a list of forest IDs with their sizes (ha).

*Matlab procedure* for allowing gap crossing

Intuitively, a bird at a forest edge should be able to assess the distance and direction to the nearest forest patch if it is within its perceptual range. GIS tools cannot limit the searching horizon so that birds only search from the edge outwards. Therefore, we developed a Matlab procedure [1], which scans only at a certain part of the horizon and defines the distance and direction to non-self patches. The procedure scans all cells in the landscape to define edge cells. An edge cell is defined as a forest cell with (a) a distance to an edge of up to one cell-size (e.g., 20 m), and (b) with an 8-cell neighbourhood, which comprises both “inner cells” (forest cells with the same patch ID) and “outer cells” (matrix cells or forest cells with a different patch ID).

For each edge cell, the program reads the general direction toward the matrix (based on the “direction” raster produced by the GIS model) and adds 43º from each side to the scanning horizon in order to depict a “visual field” to the outside. The value of 43º was chosen by trial and error as a means of scanning slightly less than 90º, to avoid immediately diagonal neighbours. It then scans the landscape within increasing scanning radii until identifying the nearest non-self forest cell within the horizon and calculates the distance and direction to this cell.

**Reference:**

1. Matlab (2009) Matlab, the language of technical computing. Version 7.8.0.347 (R2009a) ed. Boston, MA.: The MathWorks.
