# Supplementary figures and images for: Breaking Functional Connectivity into Components: A Novel Approach Using an Individual-Based Model, and First Outcomes
Source: PLoS One. 2011 Aug 1;6(8):e22355. doi: 10.1371/journal.pone.0022355 (PMC3148224; doi:10.1371/journal.pone.0022355)

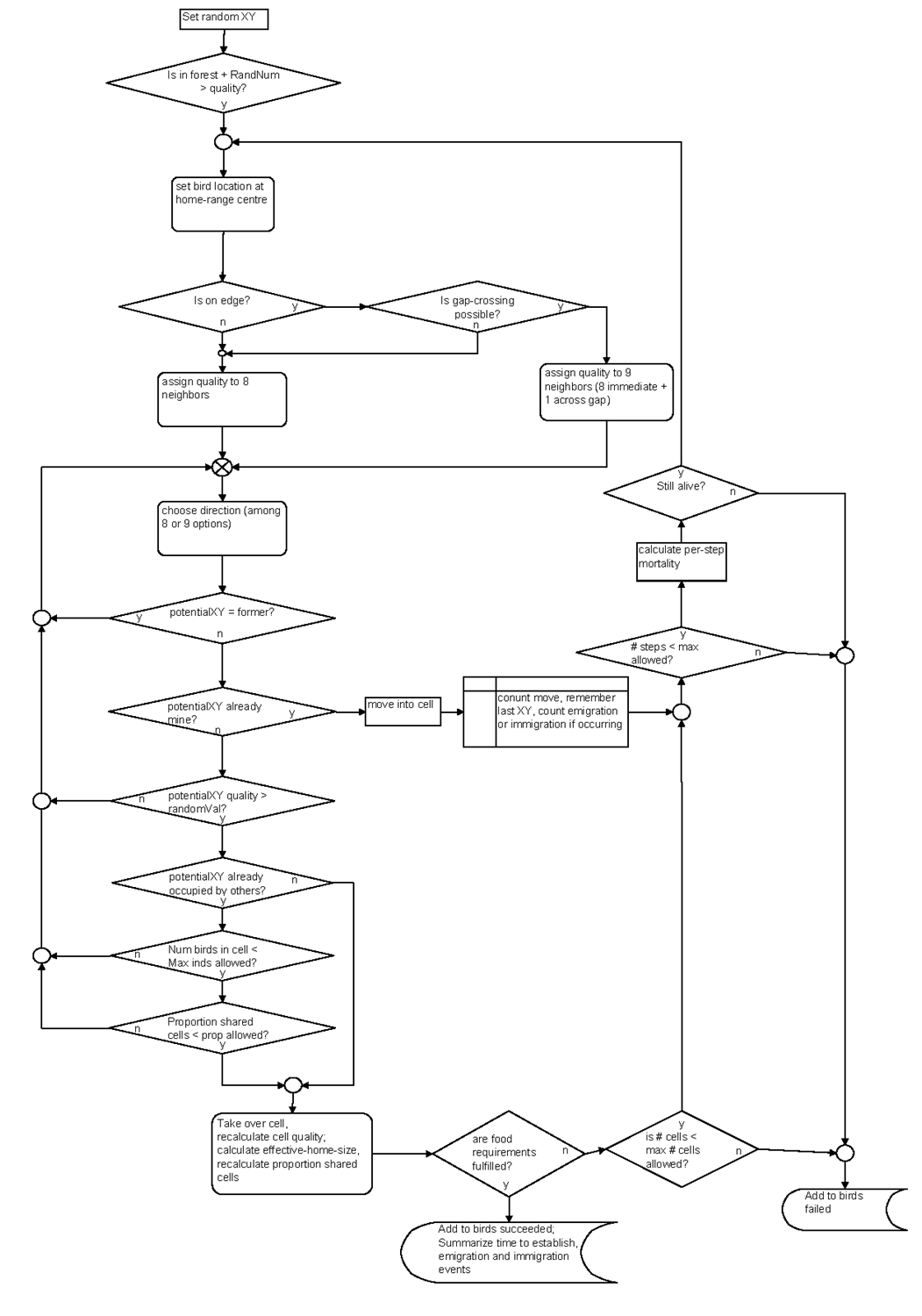

Supplement: Figure S1 — Flow chart of the process of home-range expansion. (TIF) [file pone.0022355.s004.tif]

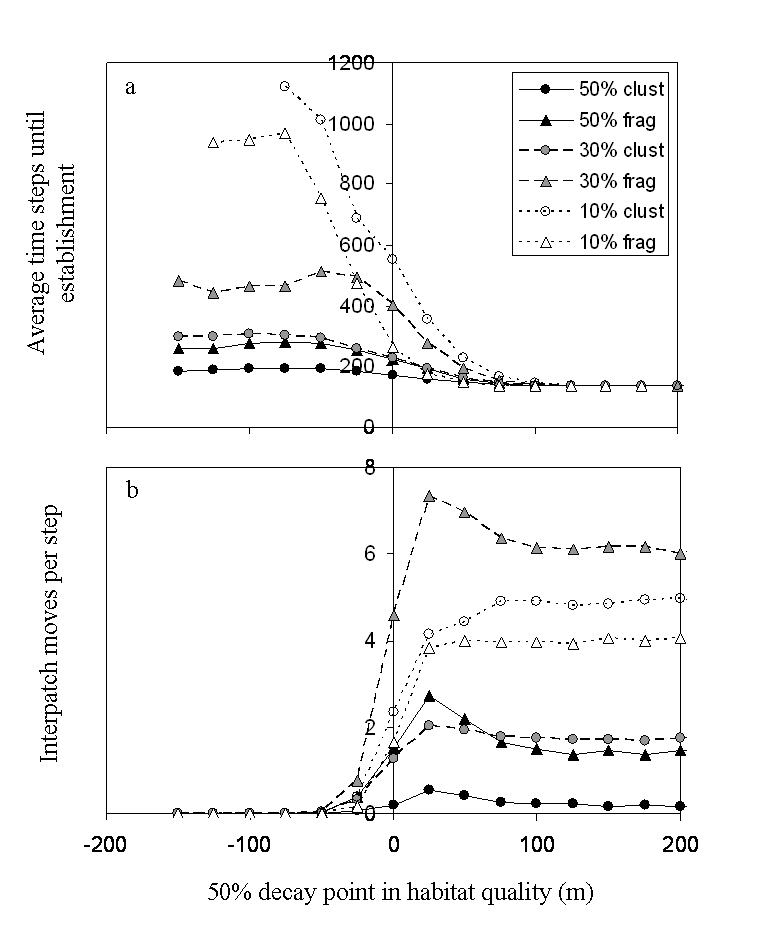

Supplement: Figure S2 — Effect of edge response and landscape structure on the time to home-range establishment. a) Number of time steps until home-range establishment, and (b) the number of interpatch movements divided by simulation duration (i.e., per step connectivity). Simulations are given for mortality per cell quality, for the six landscapes produced by the landscape generator G-RaFFe. Values in the legend represent forest cover (%) and whether the landscape was more fragmented ( = frag) or more clustered ( = clust). (TIF) [file pone.0022355.s005.tif]
